# Two new species of *Amblypsilopus* Bigot with a key to species from Taiwan (Diptera, Dolichopodidae)

**DOI:** 10.3897/zookeys.192.3265

**Published:** 2012-05-08

**Authors:** Jinjing Wang, Yajun Zhu, Ding Yang

**Affiliations:** 1 Department of Entomology, China Agricultural University, Beijing 100193, China; 2 Shanghai Entry-Exit Inspection and Quarantine Bureau, Shanghai 200135, China

**Keywords:** Diptera, Dolichopodidae, *Amblypsilopus*, new species

## Abstract

The following two new species of the genus *Amblypsilopus* Bigot from Taiwan are described: *Amblypsilopus flavellus* **sp. n.** and *Amblypsilopus ventralis* **sp. n.** One species, *Amblypsilopus crassatus* Yang, 1997, is newly reported from Taiwan. A key to the species of the genus from Taiwan is given.

## Introduction

The genus *Amblypsilopus* Bigot is a large genus in the subfamily Sciapodinae with 275 known species from the world (Bickel 1994; Yang et al.2006). There are 45 known species from China, of which 8 species occur in Taiwan (Yang et al. 2011). This genus is characterized by the following features: body usually appearing delicate with long thin legs; arista usually dorsal, shorter than head width; crossvein m-cu straight; male vertical seta reduced (Bickel 1994; Yang et al. 2011). The major references dealing with the Oriental species of *Amblypsilopus* are Becker (1922), Bickel (1994), and Yang et al.(2011). The Chinese species were reviewed by Yanget al.(2011). Here three species including two new species are added to the fauna of Taiwan. A key to the species of the genus from Taiwan is given.

## Material and methods

Types are deposited in the Entomological Museum of China Agricultural University, Beijing (CAU). The following abbreviations are used: a = anterior seta(e), acr = acrostichal seta(e), ad = anterodorsal seta(e), av = anteroventral seta(e), d = dorsal seta(e), dc = dorsocentral seta(e), ih = inner humeral seta(e), LI = fore leg, LII = mid leg, LIII = hind leg, npl = notopleural seta(e), oc = ocellar seta(e), p = posterior seta(e), pd = posterodorsal seta(e), ph = posthumeral seta(e), psa = postalar seta(e), pvt = postvertical seta(e), sa = supraalar seta(e), su = sutural seta(e), sc = scutellar seta(e), v = ventral seta(e), vt = vertical seta(e). CuAx ratio = length of m-cu / length of distal portion of CuA.

### Key to species of *Amblypsilopus* Bigot from Taiwan

**Table:** 

1	Thorax mostly metallic green	2
–	Thorax mostly yellow	*Amblypsilopus aurichalceus* (Becker)
2	Only posterior 2 dc strong, anterior dc hair-like	3
–	4–5 strong dc	10
3	Fore tibia without distinct curved posterior bristle	4
–	Fore tibia with 1 or 5–6 distinct curved posterior bristles.	6
4	Male cercus shorter than epandrium	5
–	Male cercus very long, about two times as long as epandrium.	*Amblypsilopus ignobilis* (Becker)
5	Male cercus curved, hook-like	*Amblypsilopus falcatus* (Becker)
–	Male cercus straight, finger-like	*Amblypsilopus humilis* (Becker)
6	Fore tibia with 1 pale curved posterior bristle at apical quarter; vt weak; legs elongate	7
–	Fore tibia with 5–6 distinct pale curved posterior bristles; both sexes with strong vt; legs relatively short; hind tarsomeres 3-5 flattened	*Amblypsilopus subtilis* (Becker)
7	Arista dorsal	8
–	Arista apical	9
8	Cercus broad, nearly elliptic	*Amblypsilopus mutatus* (Becker)
–	Cercus narrow, not elliptic	*Amblypsilopus crassatus* Yang
9	Wing with brown apico-anterior spot; thoracic pleuron yellow; male cercus thin, filiform, longer than epandrium	*Amblypsilopus pallidicornis* (Grimshaw)
–	Wing entirely hyaline; thoracic pleuron black except metapleuron yellow; male cercus thick, finger-like, shorter than epandrium (Fig. 1)	*Amblypsilopus flavelllus* sp. n.
10	4 strong dc; hind tarsomeres 4-5 strongly flattened	*Amblypsilopus imitans* (Becker)
–	5 strong dc; hind tarsomeres 4-5 normal	*Amblypsilopus ventralis* sp. n.

## Taxonomy

### Amblypsilopus crassatus

Yang, 1997

http://species-id.net/wiki/Amblypsilopus_crassatus

Amblypsilopus crassatus Yang, 1997: 133. Type locality: China: Zhejiang, Hangzhou.

#### Diagnosis.

Antenna yellow except first flagellomere dark brown and subrectangular. Fore tarsomere 5 distinctly flattened with lateral flags. Male cercus rather thick with ventral surface slightly concave; hypandrium rather wide.

#### Specimen examined.

1male, Taiwan: Nantou, Lienhuachi, 675 m, 2010. XI. 11, Ding Yang.

#### Distribution.

China (Henan, Hubei, Yunnan, Guizhou, Guangxi, Guangdong, Zhejiang, Fujian, Taiwan); Singapore.

#### Remarks.

This species belongs to the *Amblypsilopus triscuticatus* group (Yang et al. 2011). It is newly recorded from Taiwan.

### Amblypsilopus flavellus sp. n.

urn:lsid:zoobank.org:act:63EB632F-0262-47ED-BAF0-14AAFC47E176

http://species-id.net/wiki/Amblypsilopus_flavellus

Fig. 1

#### Diagnosis.

2 long and strong paired acr. Antenna yellow. Mesonotum with small anterolateral area including humerus and large posterolateral area including postalar callus dark yellow; metapleuron yellow. Abdomen partly yellow at base. Fore tarsomere 1 white, slightly longer than mid and hind tarsomere 1.

#### Description.

Male. Body length 4.5 mm, wing length 4.0 mm.

Head brightly metallic green with pale grey pollen. Hairs and bristles on head pale yellow; frons with 1 pale curved and slightly thick hair on posterolateral slope; 1 pvt at end of postocular line. Ocellar tubercle with 2 long strong oc and 2 posterior hairs. Antenna yellow; pedicel with circlet of short blackish apical bristles except 1 dorsal bristle and 1 ventral bristle relatively long; first flagellomere short triangular, nearly as long as wide; arista [broken apically] apical, blackish. Proboscis yellow with pale yellow hairs; palpus yellow with pale hairs and 2 brownish yellow bristles.

Thorax brightly metallic green with pale grey pollen, except mesonotum with small anterolateral area including humerus and large posterolateral area including postalar callus dark yellow; metapleuron yellow. Hairs and bristles on thorax black; 2 long strong black posterior dc and 3 pale yellow anterior hairs; 2 long and strong paired acr, 3-4 very short paired hairs anteriad; 1 short ih, 1 short ph, 1 short su, 2 sa, 1 psa, 2 npl; scutellum with 2 sc, basal pair absent. Legs yellow except fore tarsomere 1 white, mid and hind tarsomere 1 dark brown. Hairs and bristles on legs black except coxae with pale yellow hairs and brownish yellow bristles. Fore coxa with 3 bristles, hind coxa with 1 exterior bristle. Fore and mid femora thickened with narrowed apex. Fore tibia without distinct d, but with 1 long brownish posterior bristle at apical 1/5; apically with 1 av. Mid tibia with 1 a at middle, 1 ad at base and 4 pd; apically with 1 ad and 1 av. Hind tibia with row of pd and pv; apically with 1 ad and 1 av. Hind tarsomere 1 with 1 v at extreme base. Relative length ratio of tibiae and tarsomeres: LI 3.9 : 2.5 : 0.95 : 0.85 : 0.4 : 0.3; LII 4.1 : 3.2 : 0.85 : 0.6 : 0.3 : 0.2; LIII 5.7 : 2.6 : 1.0 : 0.7 : 0.4 : 0.2. Wing hyaline, veins dark brown. Vein M_1_ basally curved nearly at a right angle. Crossvein m-cu straight, CuAx ratio 1.3. Squama yellow with dark brown margin and with pale hairs. Halter dark yellow.

**Figure 1.**
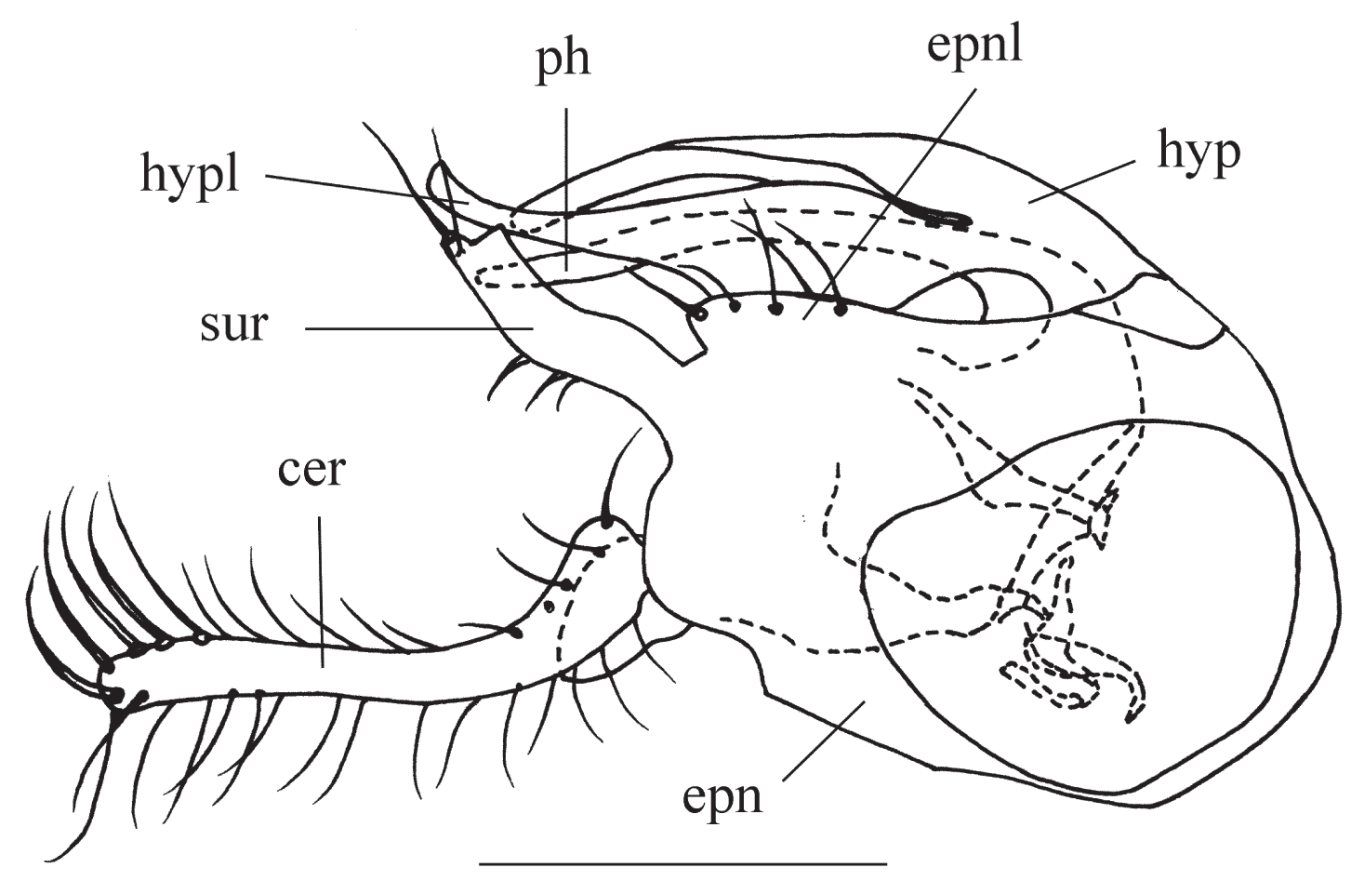
*Amblypsilopus flavellus* sp. n. Male genitalia, lateral view. Scale bar 0.25 mm. Abbreviations: **cer** cercus **epn** epandrium **epnl** epandrial lobe **hyp** hypandrium **hypl** hypandrial lateral arm **ph** phallus **sur** surstyllus.

Abdomen pale metallic green with thin pollen except segments 1-4 dark yellow or yellow with posterior margins of tergites 1-4 brown or dark brown. Hairs and bristles on abdomen black. Male genitalia (Fig. 1): Epandrium longer than wide in lateral view; epandrial lobe short, obtuse. Surstylus finger-like, slightly bent. Cercus dark yellow, shorter than epandrium, finger-like, basally slightly curved. Hypandrium apically nearly straight, with lateral arm rather thick near base.

Female. Unknown.

#### Type material.

Holotypemale, Taiwan: Kaohsiung, Nancai Mountain, 50 m, 2009. VI.

#### Distribution.

China (Taiwan).

#### Remarks.

This species belongs to *Amblypsilopus pallidicornis* group. It is similar to *C. flavicercus* Zhu *et* Yang from Hainan of China, but can be separated from it by the antenna entirely yellow, male cercus shorter than the epandrium and distinctly curved basally, and surstylus long and distinctly bent. In *C. flavicercus*, the antennal scape and flagellum are brownish at tip, male cercus is as long as the epandrium and nearly straight, and the surstylus is short and nearly straight (Yang et al. 2011).

#### Etymology.

The specific name refers to the yellow antenna.

### Amblypsilpus ventralis sp. n.

urn:lsid:zoobank.org:act:D54756C1-64E7-4123-9F61-60706038CCE4

http://species-id.net/wiki/Amblypsilpus_ventralis

Fig. 2

#### Diagnosis.

1 strong vt. 5 strong dc. 3-4 very short paired acr present only before 1 anteriormost dc. Fore femur with two rows of short v. Mid tarsomeres 4-5 slightly thickened.

#### Description.

Male.Body length 3.8-3.9 mm, wing length 3.0-3.2 mm.

Head brightly metallic green with pale grey pollen. Hairs and bristles on head black except middle and lower postocular bristles including posteroventral hairs pale yellow; frons with 1 strong vt, anteriorly without hair on posterolateral slope; 1 pvt near end of postocular line. Ocellar tubercle with 2 long strong oc and 2 posterior hairs. Antenna black; pedicel with circlet of short black apical bristles except 1 dorsal bristle and 2 ventral bristles relatively long; first flagellomere nearly trapezoid, nearly as long as wide; arista dorsal, blackish. Proboscis mostly reddish yellow with blackish hairs; palpus brownish yellow with blackish hairs and 2 black bristles.

Thorax brightly metallic green with pale grey pollen. Hairs and bristles on thorax black; 5 long strong dc; 3–4 very short, paired acr present before anteriormost dc; h indistinct, 1 ih, 1 ph, su absent, 2 sa, 1 psa, 2 npl; scutellum with two pairs of sc, basal pair very short and hair-like (about 1/5 of apical pair). Legs yellow except mid and hind coxae brown with yellow apex and tarsi dark brown from tip of fore tarsomere 1 onward. Hairs and bristles on legs black except coxae with pale yellow hairs and bristles. Fore coxa with 3 bristles, hind coxa with 1 brownish yellow exterior bristle. Fore femur thickened with two rows of v on basal 2/3 and narrowed on apical 1/3. Fore tibia ventrally slightly swollen at base, with 4 pv and one row of short dense av hairs, without distinct d; apically with 1 pd and 1 p. Mid tibia with 3 strong ad and 3 weak pd; apically with 1 ad and 1 av. Hind tibia with 4 pd and 2-3 weak pv at middle; apically with 1 ad and 1 av. Hind tarsomere 1 with 1 v at extreme base. Relative length ratio of tibiae and tarsomeres: LI 1.9 : 1.7 : 0.7 : 0.5 : 0.3 : 0.2; LII 3.2 : 2.4 : 0.9 : 0.6 : 0.2 : 0.25; LIII 4.0 : 1.5 : 1.1 : 0.6 : 0.4 : 0.2. Wing nearly hyaline, veins dark brown. Vein M_1_ basally curved nearly at a right angle. Crossvein m-cu straight, CuAx ratio 1.3. Squama yellow with dark brown margin and with pale hairs. Halter dark yellow.

**Figure 2.**
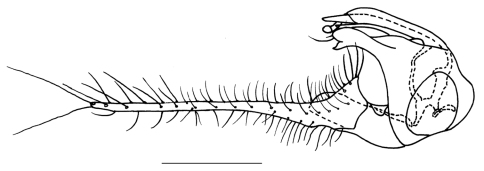
*Amblypsilopus ventralis* sp. n. Male genitalia, lateral view. Scale bar 0.25 mm.

Abdomen metallic green with thin pollen except venter and hypopygium pale metallic green. Hairs and bristles on abdomen black except those on lateral portion of tergite 1 pale yellow. Male genitalia (Fig. 2): Epandrium wider than long in lateral view; epandrial lobe indistinct. Surstylus slightly thick, apically shallowly furcated. Cercus about two times as long as epandrium, long finger-like, basally thick with subtriangular ventral process. Hypandrium distinctly bent apically, with thin lateral arm.

Female. Unknown.

#### Type material.

Holotypemale, Taiwan: Wulai, Fushan, Shuiguan Road, 2007. V. 18, Nanyi Cai. Paratype 1 male, same data as holotype.

#### Distribution.

China (Taiwan).

#### Remarks.

This species is somewhat similar to *Amblypsilopus basalis* Yang from Southern China, but can be separated from it by the antenna black, and male cercus as long as the epandrium, with the large ventral process at base. In *Amblypsilopus basalis*, the antenna is yellow, and male cercus is about two times as long as the epandrium and has the small ventral process at base (Yang 1997; Yang et al. 2011).

#### Etymology.

The specific name refers to the fore femur with two rows of short v.

## Supplementary Material

XML Treatment for Amblypsilopus crassatus

XML Treatment for Amblypsilopus flavellus

XML Treatment for Amblypsilpus ventralis
